# Determining Strategies for Constructing the Safety Supervision System by Considering Both Internal and External Safety Environments: A Case Study of X Group Corporation, China

**DOI:** 10.3390/ijerph17249486

**Published:** 2020-12-18

**Authors:** Qiaoli Wang, Xianyan Peng, Zijun Li

**Affiliations:** 1 School of Resources and Safety Engineering, Central South University, Changsha 410083, China; ql_wang@csu.edu.cn; 2 College of Environment and Resources, Fuzhou University, Fuzhou 350116, China; pengxianyan@sgpartner.cn

**Keywords:** group corporation, safety supervision strategies, internal and external safety environments, SWOT, QSPM, safety supervision system

## Abstract

Safety supervision aims to safeguard the overall interests of the corporation, and ensure its safety production together with sustainable development. It is the key to reducing accident rates, and safeguarding employees’ safety and corporate property. The establishment of safety supervision system requires specify strategies. However, it is difficult to determine such strategies in an objective manner under complex environments. Therefore, first, this paper combined an external factor evaluation matrix (EFEM) and an internal factor evaluation matrix (IFEM) to analyze the internal and external safety environments faced by X Group Corporation (XGC). Second, the strength-weakness-opportunity-threat (SWOT) approach was employed to qualitatively analyze and explore the alternative safety supervision strategies. Following this, the most attractive strategies were selected from alternatives by using the quantitative strategic planning matrix (QSPM), namely construction of the safety culture system, clarification of modes and organizational structure of safety supervision, and improvement of the safety performance evaluation system. These strategies were subsequently applied in the construction and improvement of the XGC’s safety supervision system. This study can provide reliable theoretical and methodological support for the establishment of corporations’ safety supervision systems.

## 1. Introduction

According to national laws, regulations, and the demands of safety production as well as sustainable development, it is required to strengthen the safety supervision of the group corporation. These researches on the safety supervision strategies and systems are helpful for the group corporation to control the overall levels of safety management and implement effective safety supervision processes. Moreover, they can contribute to reductions in accident rates and damage of equipment and facilities, safeguard employees’ physical safety and corporation’s property, and maintain social harmony and stability. Thus, it is very important for the group corporation to construct a systematic, reliable and objective safety supervision system. Many efforts have been made to study safety supervision system, and most of them are involved in a specified safety supervision strategy. Existing studies commonly consider safety culture, occupational health and safety management, safety management information and safety performance evaluation as key factors in building safety supervision systems. In addition, among these factors, safety culture and safety performance evaluation have received more attention.

The safety culture is a crucial factor that sets the tone for the importance of safety within an organization [1]. It refers to a set of attitudes, beliefs, and perceptions shared within natural groups [2,3], and can guide employees’ behaviors towards risk [4,5,6]. The safety culture should emphasize managers’ concerns and commitment to employees’ safety [7,8,9], mainly because employees’ perceptions of the safety system, which appear to impact employees’ behaviors and decisions in the workplace, are related to management’s commitment to safety [1]. Consequently, a commitment to safety will positively or negatively influence injury rates within corporations.

In recent years, the occupational health and safety management system has been paid increasing attention [10]. Many countries have set occupational health and safety standards for the construction of such system, and scholars have widely studied these standards. For example, the American National Standard for Occupational Health and Safety Management Systems [11] was explored by Autenrieth et al. [12]. They put forward the requirements for the participation of managers and employees. Yazdani et al., Ghahramani, and da Silva and Amaral [13,14,15] researched the Occupational Health and Safety Assessment Series 18001 (OHSAS 18001) and British Standard 8800 (BS 8800) [16,17]. The OHSAS 18001 pointed out requirements for the management of occupational health and safety (OHS) in organizations. The BS 8800 illustrated the possibilities for building up an occupational safety management system. Moreover, it had been translated into Finnish, and a corresponding website had been certified. The main purpose of these standards is to create and maintain a safe work environment for employees, to ensure their safety and health in the workplace [18].

Safety performance evaluations are conducted within an enterprise to evaluate employees’ operational behaviors and achieve work outcomes that are based on specific evaluation criteria and indicators. The evaluation results are then used to guide and improve employees’ future operations. Safety performance encompasses safety compliance and participation [19,20]. Most scholars evaluated safety performance through two dimensions: safety compliance behavior and safety participation behavior [21,22], and have explored the relationship between these two dimensions and unsafe behaviors of employees [23]. It has been proved that these two dimensions are important indicators of injuries and accidents in the process industry [24]. Safety performance evaluations comprise pre-accident assessment (proactive measurement) and post-accident assessment (reactive measurement) [25]. Proactive studies have focused on evaluating the safety climate of a region, safety culture, hazard identification and observation [26], while reactive studies have generally emphasized injury rates and compensation costs [27]. In addition, it has been found that reactive studies usually measure safety performance according to historical events or data, regardless of current safety activities [28].

Given their widespread use and close integration with business processes, information systems now have a prominent role in supporting or shaping corporate strategies [29,30]. Safety management information systems (SMIS) are combined with the elements of occupational health management system recommended by countries to develop and design [31,32]. The safety management system model in OHSAS 18001 is regarded as the macro-theoretical basis of most developed SMIS [33,34]. SMIS, developed using modern technology, are integral to the comprehensive processing of safety information relating to modern safety management, providing a key tool that supports decision making relating to safety management [35]. Therefore, safety managers can benefit greatly from the correct implementation and maintenance of SMIS [36,37].

This paper reports a case study of X Group Corporation (XGC) located in Fujian, China. The XGC is a comprehensive state-owned enterprise group focusing on new energy, new materials, medical health and finance, and involves industries such as coal, electricity, building materials, port logistics, construction and real estate, etc. It owns more than 40 wholly-owned or holding companies, including four listed ones, and is one of the top 500 enterprises in China. In order to meet the safety development needs of the XGC, it is urgent to formulate safety supervision strategies and establish a complete safety supervision system.

From the above analysis, it is apparent that previous studies on safety supervision systems have mainly focused on specific safety supervision strategies. However, the provision of objective and strategic guidance for the construction of the safety supervision system of XGC characterized by complex internal and external safety environments remains challenging. To address this challenge, an in-depth analysis of the internal and external safety environments of the X Group Corporation was performed using an internal factor evaluation matrix (IFEM) and an external factor evaluation matrix (EFEM). Next, a strengths-weaknesses-opportunities-threats (SWOT) analysis was conducted to explore alternative safety supervision strategies for XGC. Additionally, a quantitative strategic planning matrix (QSPM) was employed to select the most attractive strategies out of the alternatives. Finally, a safety supervision system was constructed, incorporating the selected strategies. Such an analysis of the strategic environment is beneficial to the formulation and selection of strategies [38]. Considering the internal and external safety environments of XGC, the strategies involved in safety supervision system can be fully taken into account through strategic decision analysis, so it provides a reliable theoretical and methodological support for the construction of the XGC’s safety supervision system.

The remainder of this paper is organized as follows. Section 2 introduces the methods used for selecting safety supervision strategies and constructing the safety performance evaluation system. Section 3 provides a detailed description of the methods applied to select the safety supervision strategies. In Section 4, based on the selection results, safety supervision system is constructed from the safety culture system, the mode and organizational structure of safety supervision, and the safety performance evaluation system. Finally, the conclusions of the study are presented in Section 5.

## 2. Method

### 2.1. Methods for the Selection of Safety Supervision Strategies

**Figure 1:**
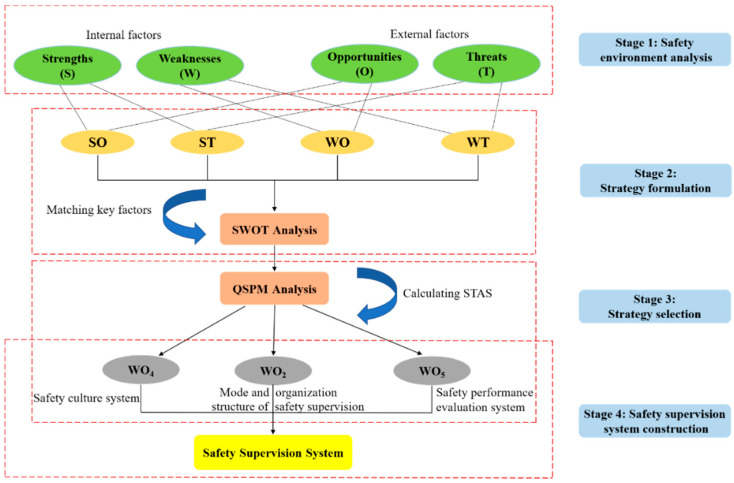
A schematic framework of proposed methods for constructing the safety supervision system.

To select reasonable and attractive strategies, an analysis of the internal and external safety environments of XGC was initially conducted, and alternative safety supervision strategies were developed based on the results of the analysis. Optimal strategies were then selected from among these alternatives and used to construct the safety supervision system of XGC. Figure 1 provides a visual depiction of the proposed methods within a schematic framework. There are two main reasons why this analytical framework was applied. On the one hand, the internal and external environmental factors of XGC can be analyzed simultaneously in the decision-making process. On the other hand, multiple groups of safety supervision strategies that were unlimited in number could be investigated at the same time. The analytical methods are described in detail in the following subsection.

#### 2.1.1. Analysis of the Internal and External Environments

Internal factor evaluation matrix (IFEM) and external factor evaluation matrix (EFEM) are usually employed to analyze how an organization is performing based on identified internal and external environments [39]. In this study, they were utilized to identify the key factors affecting the future development of corporations from the strengths and weaknesses of the internal environment along with the opportunities and threats of the external environment. Too few key factors cannot fully describe the internal and external environments of the XGC, while too many factors will make the selection of safety supervision strategy selection very difficult or even infeasible. To balance comprehensiveness and practical operability, it is suggested to control the number of key factors between 5 and 20 (15 in this study). The weights of key factors were determined according to the degree of their influence on the development, and scores were assigned to each key factor according to the effective response degree of corporations. Unreasonable setting of weights may underestimate or overestimate the importance of certain factors. To avoid the effects of unreasonable setting, both the key factors and their weights are determined according to the experience of experts. In practice, for companies that have accumulated long-term safety records, more scientific guidance can be obtained by learning knowledge from historical database. The total weighted scores of key factors were calculated by multiplying their weights by their scores. Through IFEM and EFEM analysis, the opportunities, threats, strengths and weaknesses faced by corporations in the internal and external environments can be summarized, and the corporation’s ability to deal with internal weaknesses and external risks would be assessed on the basis of the total weighted score. The sequential steps of the analyses of the IFEM and EFEM are presented below.

Step 1: Data from interviews held with senior executives and experts, and relevant materials were compiled and analyzed. We invited 12 experts, including safety managers, safety supervision and management researchers, and professional technicians, to individually select 10 strengths (opportunities) and 20 weaknesses (threats). Then, the strengths and weaknesses were sorted according to the selection count. The top 5 strengths and the top 10 weaknesses were finally identified as the key factors.

Step 2: Following consultations with experts, each key factor was assigned a weight ranging between 0 and 1.0. Specifically, each expert was invited to give an initial weight of each key factor on a ten-point scale. Then, for each key factor, we calculated the average value of initial weights after removing the highest and lowest values. Finally, the weight of each factor was obtained by normalizing the average value so that each weight is between 0 and 1 and the sum of all weights is 1. A higher weight corresponded to a higher level of importance of the key factor.

Step 3: Each key factor was assigned a score using a four-point scale, according to the effective response of corporation’s current strategies to each key factor. In IFEM (EFEM), 1 and 2 indicated main weaknesses and general weaknesses (major threats and mild threats); 3 and 4 represented general strengths and main strengths (general opportunities and major opportunities).

Step 4: The weighted score was obtained by multiplying the score of each key factor by the corresponding weight.

Step 5: The total weighted score of the internal (external) environment was calculated as the sum of all of the weighted scores. A score above a given threshold indicated that the enterprise had a healthy internal (external) environment. In this study, the score threshold was set to 2.5 [40], which is the average of the total weighted scores ranging from 1.0 to 4.0. This threshold can better distinguish healthy and unhealthy environments, and has been widely used in many application fields, such as tourism management [38,41] and waste management [42].

#### 2.1.2. Alternative Strategy Formulation

In light of the results of the IFEM and EFEM analysis, a strength (S)-weakness (W)-opportunity (O)-threat (T) (SWOT) approach was performed to enable the formulation of alternative safety supervision strategies, in which “S” (strengths) and “W” (weaknesses) represent key internal factors and “O” (opportunities) and “T” (threats) represent key external factors [43]. This method is widely applied in the areas of sustainable business management, recycling industry development, sustainable ecotourism development [42,44,45]. In this study, it was used to systematically summarize the S, W, O and T faced by the XGC. The SO, WO, ST, WT strategies will be created, when we match S, W, and O, T with each other. SWOT analysis is intended to aid organizations in developing their advantages, overcoming their weaknesses, seizing opportunities, and avoiding threats. It enables the matching of seemingly independent key factors within a comprehensive analysis, thereby ensuring that corporations’ strategies are comprehensive and scientifically grounded.

#### 2.1.3. The Selection of the Most Attractive Strategies

The quantitative strategic planning matrix (QSPM) is an important analytical tool for prioritizing different strategies formulated on the basis of from a SWOT analysis [46], thereby facilitating the selection of the most attractive safety supervision strategies. The attractiveness scores (AS) of alternative strategies were based on a consideration of whether each strategy could induce organizations to exploit external opportunities and internal advantages fully and reduce external threats and internal weaknesses as far as possible. AS was scored using a four-point scale, with attractiveness rated on a scale of 1–4. The total attraction scores (TAS) were calculated by multiplying the AS by the weights obtained from the analysis of the internal and external environments. The sum of total attraction scores (STAS) was obtained by adding all of the TAS obtained for a particular strategy. It reflected the attractiveness of strategies, resulting in the selection of strategies with higher STAS.

### 2.2. Methods for Construction of Safety Performance Evaluation System

#### 2.2.1. Construction of the Index System

The balanced scorecard (BSC) was used to determine the safety performance evaluation indexes. This method, which was proposed by American scholars [47,48], is based on information, and comprehensively considering the driving factors of corporation performance. It encompasses four dimensions: finance, customers, internal operations, and learning and growth. The importance of each dimension depends on whether the dimension itself and the selection of indexes are consistent with the XGC’s strategy. The BSC method takes the XGC’s strategy as its core and builds a platform for evaluating strategies and performance. It allows for comprehensive control of process and target management, takes account of financial and non-financial indexes, achieves a balance between short-term safety objectives and long-term strategic planning, and promotes performance management and evaluation.

#### 2.2.2. Calculation of Index Weights

The analytic hierarchy process (AHP), which is a combined qualitative and quantitative analysis method proposed by Saaty [49,50], an American professor, is widely used to calculate index weights. Applying this method, the elements relating to decision making are decomposed into different hierarchical structures, such as goals, criteria, and schemes. Subsequently, by solving the eigenvector of a judgment matrix, the weight of every element within each layer can be calculated, and the weight of the overall goal can be obtained through a process of hierarchical merging. The specific steps in this analytical procedure are outlined below.

Step 1: The structure model of a ladder hierarchy is established based on an index system. The top and bottom layers of the structure respectively correspond to the destination and scheme layers, and the middle layer corresponds to the criterion layer.

Step 2: A judgment matrix entailing pairwise comparison is established. In the model of hierarchical structure, the numbers 1–9 and their reciprocal are used as a scale for evaluating the importance of factors. In Judgment Matrix A (Equation (1)), the value $a_{ij}$ in the row *i* and the column *j* refers to the ratio of the importance of factor *i* with respect to factor *j*. Similarly, the value $a_{ji}$ is the ratio of the importance of factor *j* with respect to factor *i*, and is equal to 1/$a_{ji}$. Thus, Judgment Matrix A is obtained by comparing the relative importance of the factors at the current level to that at the upper level.

Step 3: The maximum eigenvalue ($\lambda_{max}$) and its corresponding eigenvector are obtained based on the judgment matrix. Then, the eigenvector is normalized to obtain the weight ω. To test the consistency of the judgement matrix, the consistency index (CI) and consistency ratio (CR) are introduced, as shown in Equations (2) and (3).

(1) $$A=\left[\begin{array}{ccccc} 1 & a_{12} & a_{13} & \ldots & a_{1n}\\ a_{21} & 1 & a_{23} & \ldots & a_{2n}\\ a_{31} & a_{32} & 1 & \ldots & a_{3n}\\ \ldots & \ldots & \ldots & \ldots & \ldots \\ a_{n1} & a_{n2} & a_{n3} & \ldots & a_{nn} \end{array}\right]$$

(2) $$CI=\frac{\lambda_{max}-n}{n-1}$$

(3) $$CR=\frac{CI}{RI}$$

When *CI* = 0, the judgment matrix demonstrates complete consistency. The greater the deviation degree between *CI* and 0, the more inconsistent the judgment matrix is. To further evaluate the degree of consistency, we introduce a consistency ratio (CR), which is defined as the ratio of CI to a random consistency index (RI), as shown in Table 1. If CR < 0.10, it is considered that the judgment matrix has passed the consistency test. Otherwise, the constructed judgment matrix should be modified appropriately.

## 3. Determining the Safety Supervision Strategies

In this paper, the EFEM, IFEM, SWOT and QSPM were applied in the case study of the safety supervision strategies of XGC.

### 3.1. Safety Environment Analysis

#### 3.1.1. Internal Environment

IFEM was employed to determine the key factors affecting the future development of the XGC from the strengths and weaknesses of the internal environment. Table 2 specifically summarizes and evaluates the information within XGC, including culture, education, emergency, supervision and performance respectively. The weights, scores, and weighted scores of each key internal factor are shown in this table. The total weighted score, which reflects the quality of the XGC’s internal conditions, was calculated on the basis of the weighted scores.

As shown in Table 2, 14 key internal factors (five strengths and nine weaknesses) were identified, and the total weighted score of the internal safety environment was 2.05, which is below the score threshold of healthy environment, 2.50. This shows that the internal environment is not optimistic and the weaknesses outweigh the strengths, so the XGC needs to attach great importance to this situation. Four factors had maximum weights 0.1: S_1_ was an important strength, and W_4_, W_6_ and W_10_ were the main weaknesses. Moreover, three factors, S_1_, S_2_, and S_5_, had the highest score of 4, indicating that the XGC fully exploits these factors.

#### 3.1.2. External Environment

EFEM was used to analyze the external environment of the XGC from opportunities and threats. Table 3 specifically summarizes and evaluates the information from government, law, economy, society, culture, technology and competition in the external environment. The weights, scores, and weighted scores of each key external factor are shown in this table. The weighted scores are summed to obtain the total weighted score, which was used to evaluate the ability of the XGC to grasp opportunities and avoid threats.

The EFEM analysis revealed 10 external factors (five opportunities and five weaknesses). The total weighted score of the external safety environment was 2.70, which is slightly higher than the score threshold of healthy environment (2.50), indicating that the XGC can stably cope with potential adverse effects and effectively take advantage of opportunities in the external safety environment. Moreover, O_1_, which had the maximum weight (0.15), was deemed the primary opportunity as well as the most important external key factor affecting the development of XGC. There were three other factors, O_1_, O_4_, and O_5_ that attained the highest score of 4, indicating that the XGC’s grasp of these factors is satisfactory.

### 3.2. SWOT Strategic Analysis

The SWOT analysis is an effective structured planning method used in the case of strategy formulation. Key pieces of information obtained from the EFEM and IFEM analyses were grouped into two main categories: strengths and weaknesses within the internal safety environment, and opportunities and threats within the external safety environment. As Table 4 shows, seemingly independent key factors were systematically considered, matched, and analyzed to enable the development of alternative safety supervision strategies.

The SWOT strategic analysis was performed after key internal and external factors relating to the XGC’s safety supervision had been identified and evaluated. Four strategic combinations were developed: SO, WO, WT, and ST, and a total of 11 alternative strategies were formulated. Interactions between strengths and opportunities (SO) show the good condition of the safety supervision and allow employing growth strategies. Relations between weaknesses and opportunities (WO) allow using reversal strategies. Analyses of the interactions between weaknesses and threats (WT) point to potential warning requiring the adoption of defensive strategies. Last, relations between strengths and threats (ST) could be considered as a potential for employing diversification strategies. However, because SWOT analysis tends to be qualitative and does not enable prioritization of the formulated strategies, it was necessary to perform a second quantitative analysis via QSPM and to adjust and reduce the number of achieved safety supervision strategies.

### 3.3. QSPM Strategic Selection

To provide further guidance for prioritizing alternative strategies, the QSPM was performed. This method entails evaluating and prioritizing strategies using the results of the first-stage analysis (safety environment analysis) and the second-stage analysis (SWOT strategic analysis). The left column of QSPM comprises internal and external key factors derived from the IFEM and EFEM. The top row comprises alternative strategies identified in the SWOT analysis. The specific calculations for each strategy are presented in Table 5. AS, TAS and STAS represent the attractive scores, the total attraction scores, and the sum of total attraction scores, respectively.

STAS values were employed to determine the relative attractiveness of each key factor and its associated strategy. The magnitude of differences among those values revealed the attractiveness of one strategy relative to that of others. The following STAS values of alternative strategies were computed: 6.40, 5.70, 5.60, 5.45, 5.45, 5.45, 5.45, 5.40, 5.10, 4.84 and 4.25 for WO_4_, WO_2_, WO_5_, WO_1_, WO_3_, ST_1_, ST_2_, WT_2_, WT_1_, SO_1_ and SO_2_ strategies respectively. Although 11 alternative strategies are noticeable, the three strategies (WO_4_, WO_2_, WO_5_) with higher STAS are selected from them in order to construct the safety supervision system for XGC. Hence, the safety supervision system was established from safety culture system, mode and organization structure of safety supervision and safety performance evaluation system.

## 4. The Construction of the Safety Supervision System

### 4.1. Safety Culture System

To enhance employees’ cognition and understanding of the safety concept, to cultivate employees’ awareness of safety advance, early warning and prevention, and to realize the essential safety, it is necessary to build a safety culture system and infiltrate the concept of the XGC’s safety culture into all levels of subsidiaries. Currently, there are three main problems relating to XGC’s safety culture. First, its construction is not systematic, resulting in a lack of clear direction and foresight in the construction process. Second, the extent of the safety culture’ propagation and penetration within the XGC is insufficient, and regular and systematic safety training and inspection are inadequate. Third, the terminal construction of safety culture is incomplete, and employees’ participation has been limited. The following eight dimensions were emphasized for the construction and improvement of the XGC’s safety culture to address these issues.

#### 4.1.1. The Improvement of the Safety Management System

The safety management system is the foundation of the construction of safety culture system, and it takes the implementation of safety production responsibility as the primary. Vertically, the safety management system must be refined to “what problem it is, which level is responsible and who is responsible”. Horizontally, safety responsibility should extend from the department supervising safety production to each business department, while simultaneously managing production and safety.

#### 4.1.2. The Innovation of the Safety Culture Carrier

The embodiment and dissemination of a safety culture is integrally linked to a carrier that vividly conveys that culture. Various external forms that are easily understood and recalled are collectively referred to as safety culture carriers. The carrier of the safety culture comprises several components: art, activities, education, and promotional materials to facilitate its dissemination and penetration. Specific art forms include safety evenings, photography exhibitions, and documentaries. Activities include safety knowledge contests, symposiums involving employees’ families, and emergency rescue drills. Forms of education include tertiary education, safety forums, and safety warning day. Environmental forms include safety banners and bulletin boards, safety signs, and light-emitting diode screens propagating safety knowledge.

#### 4.1.3. The Reinforcement of the Safety Training

Safety training can be directly implemented as a method of developing and improving the XGC’s safety culture. It has a significant impact on employees’ safety perceptions at the workplace, which, in turn is related to the safety climate [51]. A complete system of safety training comprises four stages. The training preparation includes the division of safety training objects, analysis of safety training needs, formulation of safety training content, and implementation of a safety training plan. The training implementation needs to pay attention to the diversification of training methods, so multimedia technology can be used to strengthen the effect of safety education and training. The training assessment is a process for testing the effectiveness of employees’ learning and should exert a certain amount of pressure on the trainees and have a certain elimination rate. The training improvement should put forward improvement suggestions and methods aiming at the problems existing in the training, by surveying or interviewing participating employees.

#### 4.1.4. The Implementation of the Safety Inspection

The implementation of the safety inspection can facilitate the development of the safety culture system. There are diverse forms of safety inspections that entail combinations of regular and irregular inspections, notifications and no notifications, and spot checks and secret checks. The XGC should disseminate the problems identified during the inspections to the subsidiaries that have not been inspected, requesting them to check each of the problems individually and report the results of the inspection and rectification process to the department that supervises safety production within a specified time frame.

#### 4.1.5. The Improvement of the Safety Information Platform

The smooth flow of information about safety supervision is a basic prerequisite for the construction of the safety culture. Therefore, the XGC should construct a safety information platform to promote the spread and penetration of the safety culture, achieve full supervision coverage, improve the management of safety production, and speed up the circulation of safety information between the XGC and its subsidiaries. The establishment of this platform could improve the accuracy and timeliness of the disseminated information and enable the XGC to achieve zero-distance management with controllable goals, assigned responsibility, and timely action. It usually comprises the following three subsystems: the basic database, daily management, and monitoring of major hazard sources and emergency rescues.

#### 4.1.6. The Attention of Grass-Roots Construction

Employees at the grassroots constitute the main force driving the construction of a safety culture. To promote the terminal construction of a safety culture and to improve employees’ participation, a mass supervision network could be established as a first step, and the safety confirmation method could then be popularized. The XGC should establish and improve measures for safety supervision and management, and launch a network platform and hotline for submitting complaints to ensure the employees’ safety supervision. Co-supervisors with different positions should be selected at the grassroots level and distributed across different teams and groups, working at varying times throughout the day to ensure adequate supervision of the safety of all employees. In addition, the safety confirmation method of finger oral [52] is a practical activity that enables the direct participation of grassroots employees in the construction of the safety culture. It can help to standardize employees’ safety behaviors and consolidate their safety awareness.

#### 4.1.7. The Implementation of the Safety Incentive Method

The formulation of a safety incentive policy facilitates the implementation of a terminal safety culture. Safety incentive methods mainly comprise economic and spiritual incentives. There are three possible incentive mechanisms: positive, negative, or a combination of positive and negative incentive mechanisms. The effects of economic incentives are short-lived, whereas the effects of spiritual incentives are enduring. While constructing a safety culture, the XGC should adhere to the combinations: economic and spiritual incentives, personal and organizational incentives, and positive and negative incentives and avoid excessive bias toward a particular incentive mode.

#### 4.1.8. The Improvement of the Safety Reporting

At the beginning of every month, the safety management personnel within the XGC’s subsidiaries should collate material on accidents, existing safety problems, and safety-related documents, inspection, and activities and report this information to the safety supervision department at the XGC. The personnel within the safety supervision department can then analyze the data and send out evaluation feedback that is returned to the subsidiaries after being identified and signed by the top safety supervisor. The subsidiaries can subsequently implement further safety management measures in light of the evaluation feedback and clarify the implementation status of the previous month’s activities when making a safety report for the following month.

### 4.2. Modes and Organizational Structure of Safety Supervision

The goal of the safety supervision modes is to divide the tasks and functions of safety supervision between the XGC and its subsidiaries, clarify what problems should and should not be managed by them, and avoid offside and blank management. From the perspective of the XGC, based on management modes and property right ties, the modes of safety supervision can be divided into the following categories: financial management and control, strategic management and control, and operational management and control.

The organizational structure for safety supervision is a hierarchical management framework established by the XGC to promote sustainable development and implement effective safety management. Within this framework, safety management authority is determined and responsibilities are allocated. However, the main problems relating to the modes and organizational structure of safety supervision have already been identified through a process of inquiry and field investigation as follows. (1) The modes of safety supervision for the XGC’s subsidiaries were unclear and not systematic, and supervision work was in a state of “doing while observing.” (2) There were only five full-time management personnel within the XGC’s safety supervision department, which was clearly inadequate, and there was no clear division of labor among them, nor requirements regarding personnel allocation. Full coverage of the safety supervision work is difficult to achieve, and work efficiency cannot be improved. Safety supervision tends to remain at a superficial level. Therefore, the following optimization methods are proposed to improve the modes and organizational structure of safety supervision.

#### 4.2.1. Supervision Modes

More than 30 wholly-owned and controlled subsidiaries of the XGC are recommended to adopt the safety supervision mode of the strategic control, while participating companies should implement the safety supervision mode of the financial control. Strategic control provides these subsidiaries with greater autonomy. The XGC could authorize its subsidiaries to implement safety management work and provide overall supervision, which is conducive to the implementation of safety production work. Moreover, the XGC head office could assist subsidiaries in many areas, such as human resources, financial resources, access to information and technology, and legal assistance. This strategy will enable the XGC to save on safety management costs and promote the sharing of available resources. When implementing the financial control mode of safety supervision, the XGC would not participate in the management of the specific production and operation processes of its subsidiaries. Instead, its focus would be on whether the subsidiaries have completed work relating to the issued financial indicators. In addition, the XGC can send management personnel to the participating subsidiaries to guide and supervise their safety management procedures.

#### 4.2.2. Organizational Structure

**Figure 2:**
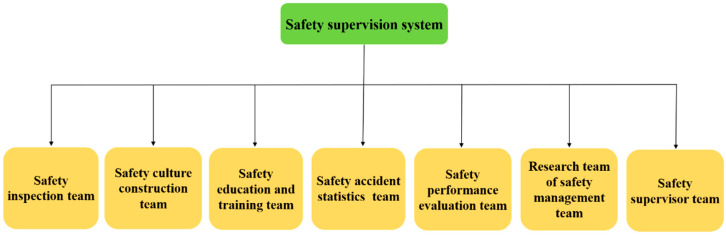
The proposed organizational structure of the safety supervision department.

In this paper, the study on the organization structure of safety supervision is actually to divide the safety management tasks and responsibilities, and to ensure the work focus of the safety supervision. The XGC should establish a safety production committee and a safety supervision department that are fully responsible for the overall safety production supervision. The safety supervision department is the core component of the organizational structure and includes both technical and management personnel, with the proportion of management personnel exceeding that of technical personnel, given that management is the key focus of this department. In addition, there is an urgent need to increase the number of full-time personnel in the safety supervision department. These staff can be recruited from the XGC’s cadre of part-time supervisors or from the wider society according to the required proportion of personnel, and a comprehensive division of labor should be implemented, as shown in Figure 2.

Figure 2 shows the components of the organizational structure. The first is the safety inspection team, whose members are responsible for inspecting the safety implementation procedures of the XGC’s subsidiaries, which covers daily, seasonal, and special inspections. The proportion of technical personnel within the safety inspection team should exceed that of the management personnel, and it is recommended to allot one technical personnel and two management personnel to each business area. The second component, safety culture, is mainly conceptualized in the form of a safety culture carrier, and its basic guarantee is safety management rules and regulations. Therefore, the main tasks of members of the safety culture construction team are to refine safety management rules and regulations, and develop a safety culture carrier. This team mainly comprises management personnel, with one or two properly equipped technicians. The third component is the safety education and training team, which is responsible for organizing and arranging safety education and training for the principal heads, safety production supervisors, safety management personnel, and emergency rescue personnel. It comprises management personnel and properly equipped technical personnel. The fourth component is the safety accident statistics team, which is responsible for compiling statistics relating safety accidents, summarizing and analyzing the causes of accidents, and collecting and sorting out the various kinds of safety information reported by subsidiaries. This team is mainly composed of management personnel. The safety performance evaluation team, which is the fifth component, focuses on process and result evaluations. This team could help to construct an effective evaluation mechanism for the XGC and promote the implementation of safety management between subsidiaries and industrial departments. This team mainly comprises management personnel. The sixth component is the team researching safety management systems, which can be composed of the leaders of other teams. Its focus is on identifying and learning domestic as well as international best practices and concepts of safety management. Most of the team members are management personnel. The final component of the organizational structure is a team of safety supervisors, who are sent by the safety supervision department to the subsidiaries to guide and inspect their safety management work. The members of this team, who are mainly technical personnel, regularly report on the safety production work to the XGC.

### 4.3. The Safety Performance Evaluation System

The goal of safety performance evaluation is to improve the XGC’s safety performance and subsequently achieve the purpose of reducing investments in safety production by identifying and developing the abilities of the employees and the cooperative skills of the teams [53]. Safety performance evaluation system is based on the key factors to select evaluation indexes and to define the weights of each index and, to evaluate employees’ operational behaviors and work results. High-performing subsidiaries should be rewarded, and punitive measures should be taken against those that do not perform well according to the results of the evaluation.

#### 4.3.1. Determining Indexes

The balanced scorecard (BSC) was used to establish the safety performance evaluation index system on the basis of four dimensions: safety finance, external safety management, internal safety management, learning and growth, as shown in Table 6.

#### 4.3.2. Determining Weights

We employ the analytic hierarchy process (AHP) introduced in Section 2.2.2 to quantify the weights of different safety performance evaluation indexes. The method is widely used in group decision making applications due to its ability to decomposes the complex decision problem into a hierarchy of more easily comprehended sub-problems. A potential problem in its practical use is the complex computations caused by the large order of the judgment matrix. In this case, computer programming software (e.g., MATLAB) can be used to improve the computational performance of the AHP method. Table 7, Table 8 and Table 9 outline the specific process used to determine index weights at three levels.

(1) The third-level index weights

The safety investment corresponding to the third-level index weights were calculated as follows.

With the help of MATLAB software, the weight vector (ω) and the consistency ratio (CR) of judgment matrix were calculated using Equations (1)–(3) shown in Section 2.2.2, Their respective values were as follows: ω = (0.4955, 0.1347, 0.0380, 0.2636, 0.0682), and *CR* = 0.0711. As the value of *CR* was less than 0.1, the judgment matrix passed the consistency test.

Likewise, by comparing the indexes in pairs, constructing the judgement matrices and calculating the CI or CR, it can be concluded that the judgment matrices of safety benefit, safety image of the XGC, stakeholder, basic management, equipment and facilities management, emergency rescue management, hidden trouble detection and control, major hazard source management, operation safety, safety accident management, occupational health management, safety management organizations, safety education and training, safety regular meeting and employee literacy have all passed the consistency test. Furthermore, the weight vectors of all of the judgement matrices can be also calculated, so the third-level index weights are determined (see Table 6).

(2) The second-level index weights

The safety finance corresponding to the second-level index weights were calculated as follows.

Equations (1)–(3) were used to calculate the weight vector (ω) and the consistency index (CI) of the judgment matrix, and the following values were obtained: ω = (0.7500, 0.2500), and *CI* = 0. Given that *CI* = 0, the judgment matrix was deemed to be completely consistent.

Following the same calculation steps shown above, the judgment matrix constructed for the external safety management index (CI = 0) was found to have complete consistency, and the judgment matrix constructed for the internal safety management and learning and growth indexes (CR < 0.1) passed the consistency test. Second-level index weights were obtained by calculating the weight vectors of the judgment matrices (see Table 6).

(3) The first-level index weights

The first-level index weights of safety performance index system were calculated as follows.

The weight vector ω and the consistency ratio (CR) of judgment matrix were calculated with the help of the MATLAB software using Equations (1)–(3). The following values were obtained:

ω = (0.1998, 0.0781, 0.5222, 0.1998) and *CR* = 0.0161. Given that *CR* < 0.1, the judgment matrix was deemed to have passed the consistency test.

Finally, according to the determined indexes and the calculated weight of each index, the index evaluation system of safety performance of the XGC (see Table 6) was established. The final score for the safety performance evaluation can be calculated after the experts have assigned a score to each index.

## 5. Conclusions

This case study served to provide reliable strategic guidance for the construction of a safety supervision system for X Group Corporation (XGC). EFEM and IFEM were employed to analyze the external and internal safety environment faced by the XGC. Accordingly, 10 key external factors (five opportunities and five threats) and 14 key internal factors (five strengths and nine weaknesses) that would affect the future development of the XGC were identified. The score of 2.70 in EFEM indicates the XGC could reduce potential threats and fully exploit available opportunities. However, the score of 2.05 in IFEM showed that the XGC’s production conditions were not satisfactory. Based on these analysis results, the SWOT analysis was performed, leading to the formulation of 11 alternative safety supervision strategies, but it tended to qualitative analysis. Therefore, a qualitative analysis method QSPM was applied to select the most attractive safety supervision strategies from alternatives. Subsequently, the three most attractive strategies were determined, which included construction of the safety culture system, clarification of modes and organizational structure of safety supervision, and improvement of the safety performance evaluation system. These strategies were fully implemented in the construction of the XGC’s safety supervision system, as described in Section 4 of this paper.

In conclusion, EFEM and IFEM were found to be effective methods for identifying positive and negative factors affecting the XGC’s development. Furthermore, analytical tools, such as SWOT and QSPM, are useful for formulating and optimizing safety supervision strategies. The research model and programs described in this article will be constantly revised and updated, thus facilitating future in-depth studies aimed at identifying appropriate safety supervision strategies.

The methodology can be widely used for strategic decision-making of corporations. The factors and weights determined in this study can serve as a preliminary reference for the construction of the safety supervision system of corporations with similar background to XGC. However, they should be refined according to specific situation (strengths, weaknesses, opportunities and threats) of a target corporation. Although the experience of experts can provide certain guidance for selecting appropriate key factors and weights, the subjectivity should be reduced in the future.

## Figures and Tables

**Table 1:** Random consistency index (RI).

*n*	1	2	3	4	5	6	7	8	9	10	11	12	13	14
*RI*	0	0	0.52	0.89	1.12	1.24	1.36	1.41	1.46	1.49	1.52	1.54	1.56	1.58

**Table 2:** Internal factor evaluation matrix (IFEM) analysis.

Key Internal Factors	Weight	Score	Weighted Score
**Strengths**			
S_1_ The XGC’s leaders attach importance to safety supervision.	0.10	4	0.40
S_2_ The Safety management personnel of the XGC have solid professional knowledge and rich experience in safety management.	0.05	4	0.20
S_3_ Safety management organization of the XGC is complete;	0.05	3	0.15
S_4_ Subsidiaries have a certain safety management foundation	0.05	3	0.15
S_5_ Safety investment has performed well.	0.05	4	0.20
**Weaknesses**			
W_1_ The functional orientation of the XGC and subsidiaries is unclear	0.10	2	0.20
W_2_ The safety supervision chain is too long.	0.05	2	0.10
W_3_ The concept of safety supervision is backward.	0.05	2	0.10
W_4_ The division of safety management responsibilities is unclear.	0.10	1	0.10
W_5_ The safety supervision information platform is imperfect.	0.05	1	0.05
W_6_ The safety culture system is not perfect.	0.10	1	0.10
W_7_ The relevant safety management system is not perfect.	0.05	1	0.05
W_8_ The Safety management personnel of the XGC are not equipped enough.	0.05	2	0.10
W_9_ The safety performance evaluation system is not perfect.	0.05	1	0.05
W_10_ The safety management level of subsidiaries is uneven.	0.10	1	0.10
(1) Safety education and training have not been fully implemented. (2) The setup of the management organization is unreasonable. (3) Some employees have limited educational level, professional knowledge and safety awareness. (4) The construction of safety standardization is a mere formality, paying attention to the superficial things. (5) Emergency plan and emergency drill have not been implemented; (6) Accident handling is not timely and not in place. (7) Risk identification, hidden danger investigation, and anti-three violations activities are not thoroughly implemented. (8) Some subsidiary leaders do not pay enough attention to safety.			
**Total**		2.05	

**Table 3:** External factor evaluation matrix (EFEM) analysis.

Key External Factors	Weight	Score	Weighted Score
**Opportunities**			
O_1_ The state and society attach great importance to the safety production of enterprises, which promotes the progress of safety supervision of the XGC.	0.15	4	0.60
O_2_ The development of science and technology provides technological support for the safe production of the XGC.	0.10	3	0.30
O_3_ Safety management theories keep continuous improvement, which provides theoretical support for safety management work.	0.10	3	0.30
O_4_ Safety standardization construction brings opportunities for XGC to create good safety climate.	0.10	4	0.40
O_5_ The strategy of “enterprises going out” provides an opportunity to realize the essential safety of XGC.	0.10	4	0.40
**Threats**			
T_1_ Coal and construction are high-risk industries, which bring challenges to safety management.	0.10	1	0.10
T_2_ The global economy is in a downturn, and the XGC is facing overcapacity in some industries and industrial restructuring, which creates new difficulties for safety supervision.	0.10	2	0.20
T_3_ Safety has become the foundation and premise of the XGC’s sustainable development.	0.10	2	0.20
T_4_ Safety laws and regulations system in China are not perfect, and safety management is prone to loopholes.	0.10	1	0.10
T_5_ A safe, healthy and hygienic working environment is the basic requirement for ensuring safe production and optimizing the structure of human resources.	0.05	2	0.10
**Total**			2.70

**Table 4:** Strength-weakness-opportunity-threat (SWOT) matrix analysis.

	Key Internal Factors	Strengths (S)	Weaknesses (W)
Key External Factors		S_1_, S_2_, S_3_, S_4_, S_5_	W_1_, W_2_, W_3_, W_4_, W_5_, W_6_, W_7_, W_8_, W_9_, W_10_
**Opportunities(O)**		**SO Strategies**	**WO Strategies**
O_1_, O_2_, O_3_, O_4_, O_5_		1. Popularize safety production science and technology (S_1_, S_3_, S_5_, O_1_, O_2_, O_4_) 2. Adopt scientific safety management theories and rich safety management experience (S_2_, S_4_, O_3_, O_5_)	1. Construct the responsibility system of safety supervision (W_1_, W_4_, W_7_, O_1_, O_3_, O_4_, O_5_) 2. Clarify mode and organization structure of safety supervision (W_1_, W_2_, W_3_, W_8_, O_1_, O_3_, O_4_, O_5_) 3. Improve safety supervision information platform (W_2_, W_5_, W_8_, W_10_, O_1_, O_2_) 4. Implement construction of safety culture system (W_6_, W_7_, W_10_, O_1_, O_4_, O_5_) 5. Improve the safety performance evaluation system (W_3_, W_4_, W_7_, W_9_, O_1_, O_3_, O_4_, O_5_)
**Threats (T)**		**ST Strategies**	**WT Strategies**
T_1_, T_2_, T_3_, T_4_, T_5_		1. Improve the safety risk prevention and control system (S_1_, S_2_, S_3_, S_4_,S_5_, T_1_, T_2_, T_3_, T_4_) 2. Implement occupational health standardization construction (S_1_, S_2_, S_3_, S_4_, S_5_, T_3_, T_5_)	1. Improve the professional level and skills of safety management personnel (W_8_, W_9_, W_10_, T_1_, T_2_) 2. Establish and improve safety rules and regulations (W_4_, W_7_, T_3_, T_4_, T_5_)

**Table 5:** Quantitative strategic planning matrix (QSPM) analysis.

Key Factors		Weight	Alternative Strategies																					
			SO_1_		SO_2_		WO_1_		WO_2_		WO_3_		WO_4_		WO_5_		ST_1_		ST_2_		WT_1_		WT_2_	
			AS	TAS	AS	TAS	AS	TAS	AS	TAS	AS	TAS	AS	TAS	AS	TAS	AS	TAS	AS	TAS	AS	TAS	AS	TAS
Strength	S_1_	0.10	4	0.40	2	0.20	3	0.30	3	0.30	4	0.40	3	0.30	3	0.30	2	0.20	2	0.20	2	0.20	3	0.30
	S_2_	0.05	3	0.15	4	0.20	2	0.10	4	0.20	2	0.10	4	0.20	4	0.20	3	0.15	3	0.15	3	0.15	3	0.15
	S_3_	0.05	3	0.15	2	0.10	2	0.10	4	0.20	2	0.10	3	0.15	3	0.15	3	0.15	3	0.15	3	0.15	2	0.10
	S_4_	0.05	3	0.15	3	0.15	2	0.10	3	0.15	2	0.10	4	0.20	3	0.15	3	0.15	3	0.15	3	0.15	3	0.15
	S_5_	0.05	4	0.20	2	0.10	3	0.15	3	0.15	4	0.20	4	0.20	3	0.15	3	0.15	3	0.15	3	0.15	2	0.10
Weaknesses	W_1_	0.05	1	0.05	2	0.10	3	0.15	2	0.10	1	0.05	4	0.20	3	0.15	3	0.15	3	0.15	3	0.15	4	0.20
	W_2_	0.05	3	0.15	3	0.15	3	0.15	4	0.20	4	0.20	4	0.20	4	0.20	4	0.20	4	0.20	3	0.15	2	0.10
	W_3_	0.05	2	0.10	4	0.20	3	0.15	3	0.15	3	0.15	3	0.15	3	0.15	3	0.15	3	0.15	3	0.15	3	0.15
	W_4_	0.05	1	0.05	1	0.05	3	0.15	3	0.15	2	0.10	3	0.15	2	0.10	2	0.10	2	0.10	2	0.10	1	0.05
	W_5_	0.10	1	0.10	1	0.10	4	0.40	3	0.30	2	0.20	3	0.30	3	0.30	3	0.30	3	0.30	2	0.20	3	0.30
	W_6_	0.05	3	0.15	3	0.15	2	0.10	3	0.15	3	0.15	3	0.15	3	0.15	3	0.15	3	0.15	2	0.10	1	0.05
	W_7_	0.10	2	0.20	3	0.30	4	0.40	3	0.30	2	0.20	4	0.40	3	0.30	3	0.30	3	0.30	3	0.30	3	0.30
	W_8_	0.05	2	0.10	2	0.10	4	0.20	2	0.10	4	0.20	3	0.15	3	0.15	3	0.15	3	0.15	3	0.15	4	0.20
	W_9_	0.05	3	0.15	3	0.15	2	0.10	4	0.20	2	0.10	3	0.15	3	0.15	3	0.15	3	0.15	2	0.10	2	0.10
	W_10_	0.05	1	0.05	2	0.10	3	0.15	2	0.10	3	0.15	3	0.15	4	0.20	3	0.15	3	0.15	3	0.15	1	0.05
	W_11_	0.10	3	0.30	3	0.30	3	0.30	3	0.30	3	0.30	4	0.40	3	0.30	3	0.30	3	0.30	3	0.30	3	0.30
Opportunities	O_1_	0.15	2	0.30	2	0.30	3	0.45	2	0.30	3	0.45	3	0.45	2	0.30	3	0.45	3	0.45	3	0.45	3	0.45
	O_2_	0.10	4	0.40	1	0.10	2	0.20	2	0.20	4	0.40	3	0.30	3	0.30	3	0.30	3	0.30	1	0.10	1	0.10
	O_3_	0.10	1	0.10	3	0.30	3	0.30	3	0.30	2	0.20	3	0.30	2	0.20	2	0.20	2	0.20	2	0.20	3	0.30
	O_4_	0.10	3	0.30	1	0.10	3	0.30	2	0.20	3	0.30	3	0.30	3	0.30	3	0.30	3	0.30	3	0.30	3	0.30
	O_5_	0.10	3	0.30	2	0.20	3	0.30	4	0.40	3	0.30	4	0.40	3	0.30	2	0.20	2	0.20	3	0.30	4	0.40
Threats	T_1_	0.10	3	0.30	3	0.30	2	0.20	3	0.30	4	0.40	3	0.30	3	0.30	3	0.30	3	0.30	3	0.30	3	0.30
	T_2_	0.10	2	0.20	1	0.10	1	0.10	2	0.20	2	0.20	2	0.20	1	0.10	1	0.10	1	0.10	1	0.10	2	0.20
	T_3_	0.10	3	0.30	2	0.20	2	0.20	3	0.30	3	0.30	3	0.30	3	0.30	3	0.30	3	0.30	3	0.30	3	0.30
	T_4_	0.10	1	0.10	1	0.10	3	0.30	3	0.30	1	0.10	3	0.30	3	0.30	3	0.30	3	0.30	3	0.30	4	0.40
	T_5_	0.05	2	0.10	2	0.10	2	0.10	3	0.15	2	0.10	2	0.10	2	0.10	2	0.10	2	0.10	2	0.10	1	0.05
STAS				4.85		4.25		5.45		5.70		5.45		6.40		5.60		5.45		5.45		5.10		5.40

**Table 6:** Safety performance evaluation index system.

Safety performance index system (U)	**First-Level Index**	**Weight**	**Second-Level Index**	**Weight**	**Three-Level Index**	**Weight**
	Safety finance U_1_	0.1998	Safety investment U_11_	0.7500	①Cost of safety technical measures U_111_ ②Cost of industrial hygiene measures U_112_ ③Cost of Safety education and training U_113_ ④Cost of labor protection necessities U_114_ ⑤Cost of daily safety management U_115_	0.4955 0.1347 0.0380 0.2635 0.0682
			Safety benefit U_12_	0.2500	①Reduction of accident losses U_121_ ②Reduction of production losses U_122_ ③Reduction of property losses U_123_	0.4286 0.4286 0.1429
	External safety management U_2_	0.0781	Safety image of the XGC U_21_	0.7500	①Social satisfaction with XGC’s safety U_211_ ②Rank and position in the same industry U_212_	0.5000 0.5000
			Stakeholders U_22_	0.2500	①Safety management of stakeholder U_221_ ②Management and personnel qualifications U_222_	0.7500 0.2500
	Internal safety management U_3_	0.5222	Basic management U_31_	0.1093	①Annual safety targets U_311_ ②safety commitment U_312_ ③Safety strategic planning U_313_ ④Safety laws and regulations U_314_	0.0819 0.2346 0.2346 0.4488
	Internal safety management U_3_	0.5222	Equipment and facilities management U_32_	0.0458	①Equipment safety management U_321_ ②Special operation equipment management U_322_ ③Safety protection equipment management U_323_	0.2000 0.2000 0.6000
			Emergency rescue management U_33_	0.0458	①Emergency organization U_331_ ②Emergency plan U_332_ ③Emergency training and learning U_333_ ④Emergency equipment and materials U_334_ ⑤Emergency drill U_335_ ⑥Emergency response U_336_ ⑦Emergency recovery U_337_	0.0999 0.0606 0.1799 0.0999 0.1799 0.3398 0.0401
			Hidden trouble detection and control U_34_	0.1093	①Hidden trouble detection scheme U_341_ ②Hidden trouble detection U_342_ ③Hidden trouble control U_343_ ④Forecast and precaution U_344_ ⑤Hidden trouble information files U_345_	0.4447 0.2029 0.2029 0.0965 0.0530
			Major hazard source management U_35_	0.2551	①Identification and assessment of major hazard sources U_351_ ②Records and Files of major hazard sources U_352_ ③Supervision and management of major hazard sources U_353_	0.4286 0.4286 0.1429
			Operation safety U_36_	0.0458	①Site management and process control U_361_ ②Safety signs setting U_362_ ③Operation behaviors management U_363_	0.6370 0.1047 0.2583
			Safety accident management U_37_	0.0244	①Accident pre-control index U_371_ ②Accident investigation and handling U_372_ ③Accident statistics and analysis U_373_ ④Accident files U_374_ ⑤Accident report learning U_375_	0.2440 0.1069 0.0619 0.0619 0.5253
			Occupational health management U_38_	0.1093	①Occupational hazard management U_381_ ②Occupational health monitoring U_382_ ③Employees’ health surveillance U_383_	0.2000 0.2000 0.6000
			Safety management organizations U_39_	0.2551	①personnel allocation U_391_ ②Institutional responsibility U_392_	0.2500 0.7500
	Learning and growth U_4_	0.1998	Safety education and training U_41_	0.2583	①The plan of safety education and training U_411_ ②The process of safety education and training U_412_ ③The results of safety education and training U_413_	0.1047 0.6370 0.2583
			Safety regular meeting U_42_	0.1047	①Contents of regular safety meetings U_421_ ②Numbers of regular safety meetings U_422_ ③Attendance rate of employees U_423_	0.6370 0.1047 0.2583
			Employee literacy U_43_	0.6370	①The style of leaders U_431_ ②Professional level of management U_432_ ③Safety awareness of operators U_433_	0.1047 0.2583 0.6370

**Table 7:** Judgment matrix of the safety investment.

	U_111_	U_112_	U_113_	U_114_	U_115_
U_111_	1	5	7	3	6
U_112_	1/5	1	5	1/3	3
U_113_	1/7	1/5	1	1/6	1/3
U_114_	1/3	3	6	1	5
U_115_	1/6	1/3	3	1/5	1

**Table 8:** Judgment matrix of the safety finance.

	U_11_	U_12_
U_11_	1	3
U_12_	1/3	1

**Table 9:** Judgment matrix of the safety performance evaluation index.

	U_1_	U_2_	U_3_	U_4_
U_1_	1	3	1/3	1
U_2_	1/3	1	1/5	1/3
U_3_	3	5	1	3
U_4_	1	3	1/3	1
